# Long-term follow-up of a patient with 5q31.3 microdeletion syndrome and the smallest *de novo* 5q31.2q31.3 deletion involving *PURA*

**DOI:** 10.1186/s13039-015-0193-9

**Published:** 2015-11-14

**Authors:** Maria Clara Bonaglia, Nicoletta Zanotta, Roberto Giorda, Grazia D’Angelo, Claudio Zucca

**Affiliations:** Cytogenetics Laboratory, Scientific Institute IRCCS E. Medea, Bosisio Parini, LC Italy; Unit of Clinical Neurophysiology, Scientific Institute IRCCS E. Medea, Bosisio Parini, LC Italy; Molecular Biology Laboratory, Scientific Institute IRCCS E. Medea, Bosisio Parini, LC Italy; Neuromuscular Disorders Unit, Scientific Institute IRCCS E. Medea, Bosisio Parini, LC Italy

**Keywords:** 5q31.3 microdeletion syndrome, Array-CGH, Neurodevelopmental phenotype, *PURA* gene, 5q31.2q31.3 deletion

## Abstract

**Background:**

Purine-rich element binding protein A (*PURA*, MIM 600473), is considered the crucial phenocritical gene for an emerging 5q31.3 microdeletion syndrome. To date, at least seven affected individuals with overlapping 5q31.2q31.3 deletions, varying in size from 2.6 to 5 Mb, have been reported sharing neurologic features such as severe developmental delay, neonatal hypotonia, early feeding difficulties, respiratory distress and EEG abnormalities. The recent finding that *de novo PURA* point mutations are indeed sufficient to cause the severe neurological symptoms also observed in patients with 5q31.2q31.3 deletion further reinforces the gene’s causative role in 5q31.3 microdeletion syndrome.

**Case presentation:**

The present patient, aged 26 years, is the oldest reported individual and carries the smallest de novo 5q31.2q31.3 microdeletion encompassing *PURA* (360 kb). Her clinical history summarizes the mainly neurodevelopmental phenotype described in children with 5q31.3 microdeletion syndrome. In addition, our patient exhibited a remarkable deterioration of clinical symptoms, starting at the beginning of adolescence, pubertal delay and primary amenorrhea. While epileptic seizures were successfully treated during her life, feeding problems showed a poor outcome, her respiratory problems increased and eventually became severe enough to cause her death.

**Conclusion:**

The clinical and molecular findings reported here provide further evidence that 5q31.3 microdeletion syndrome is a clinically discernible *PURA*-related disorder and describe the previously unreported natural evolution of the disease in a 26 years old patient.

## Background

The 5q31.3 microdeletion syndrome is an emerging condition characterized by severe developmental delay, neonatal hypotonia, early feeding difficulties, early-onset seizures, respiratory distress and neuroimaging abnormalities [1–4]. To date at least seven affected children carrying 5q31.2q31.3 deletions, varying in size from 2.6 to 5 Mb, have been reported [1–4].

The minimal deletion region of ~101 Kb [5] between all published patients [1–4] harbors three genes: purine-rich element binding protein A (*PURA*), IgA-inducing protein (*IGIP*,) and cysteine-rich transmembrane module containing 1 (*CYSTM1*) [3]. *PURA*, encoding activator protein Pur- α (MIM 600473), has been proposed as the phenocritical gene [1] based on its role in neuronal development [6, 7]. The recent finding that *de novo PURA* point mutations are indeed sufficient to cause the same severe neurological symptoms observed in patients with 5q31.3 deletion further reinforces *PURA*’s causative role in 5q31.3 microdeletion syndrome [5, 8]. The present patient, aged 26 years, is the oldest reported individual and carries the smallest *de novo* 5q31.2q31.3 microdeletion encompassing *PURA* (360 kb). To our knowledge, this is the first report of the longitudinal medical history of a patient with *PURA*-related disorder.

## Case presentation

**Fig. 1:**
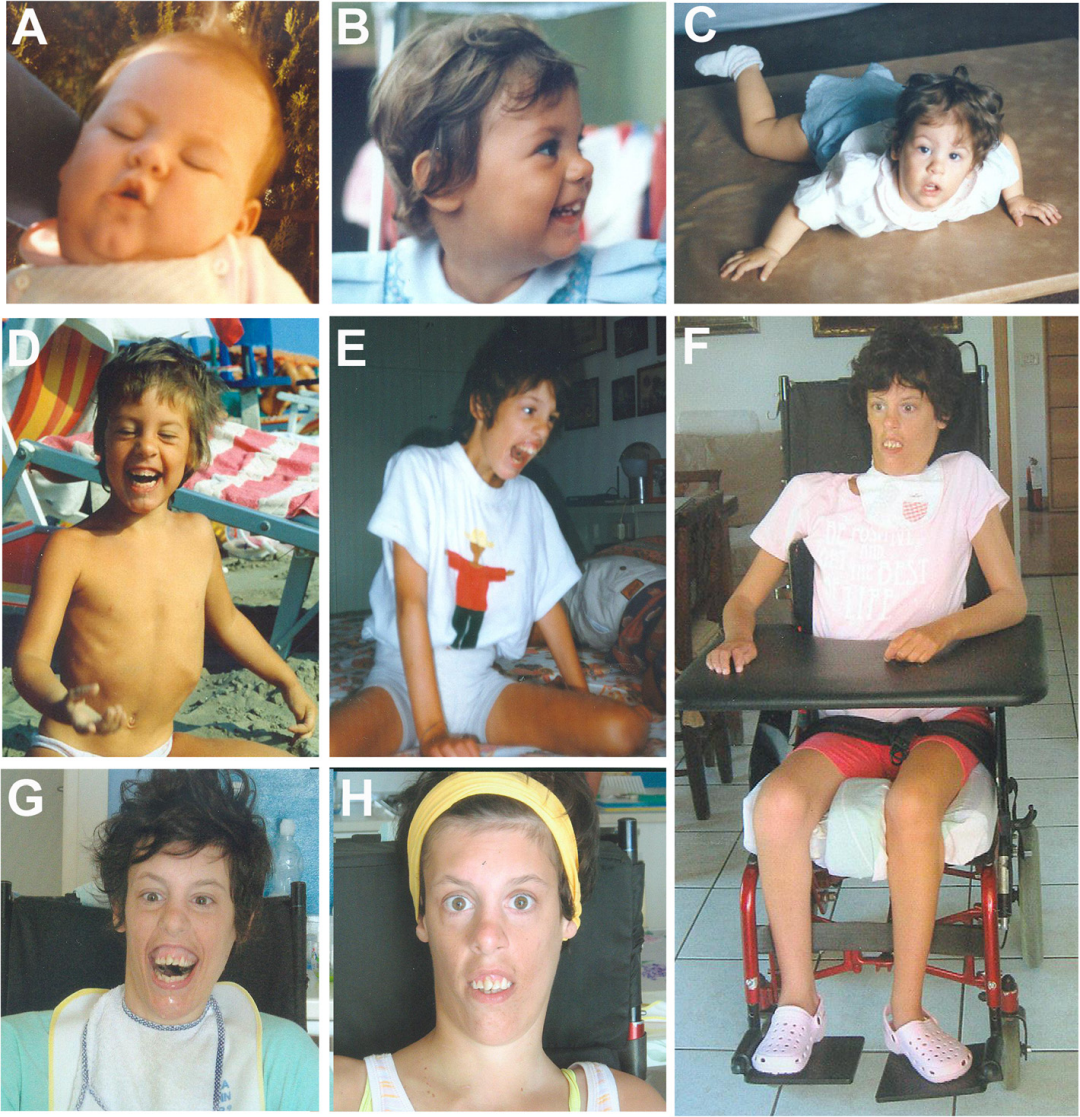
Photographs of the patient at the age of 9 months (**a**) 2 years: lateral (**b**) and frontal views (**c**) 5 years (**d**) 13 years (**e**) and 26 years (**f**, **g**, **h**). Note the long face, anteverted nares, hypertelorism, open-tended mouth and myopatic face (**a**-**h**), abnormal dentition (oversized and overlapping incisors) and gum hypertrophy (**f**-**h**)

**Fig. 2:**
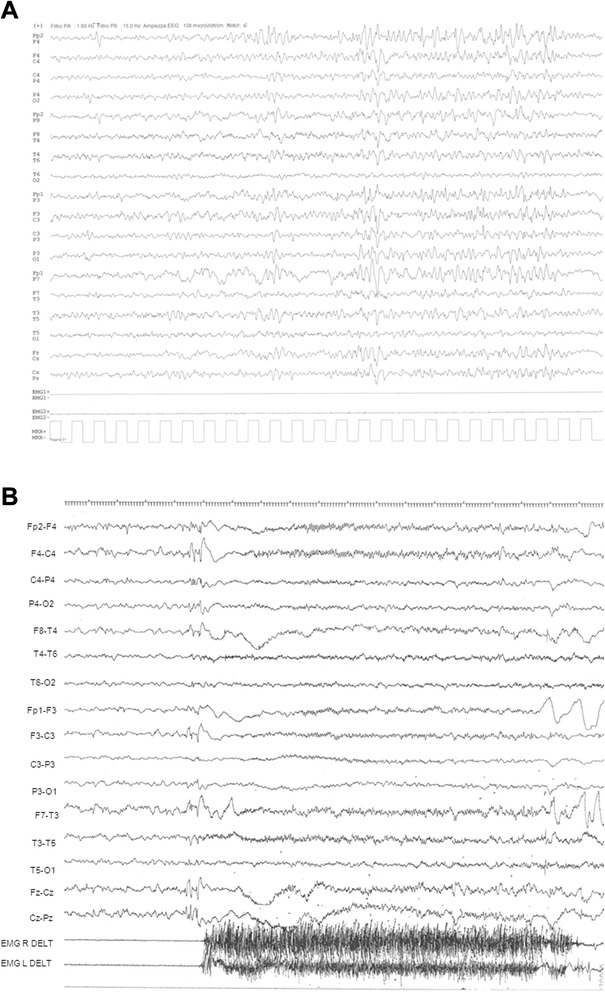
Representative EEG recordings: **a** Ictal EEG pattern (recorded at the age of 20 years during drowsiness). An EEG flattening closely related to low-voltage rapid discharge was recorded prevalently on frontal regions. EMG showed deltoid hypertonia. **b** Intercritical EEG pattern (recorded at the age of 26 years during drowsiness). The record shows irregular background activity. Slow abnormalities and spike-waves complexes, prevalent on anterior regions, were more evident during drowsiness and sleep

The proband is a female (Fig. 1a–f), the third child of healthy non-consanguineous parents aged 38 (father) and 34 (mother) years at her birth. Delivery was at 40 weeks of gestation. Birth weight was 3700 g. Length, OFC and Apgar scores were not available. At birth she was admitted to the neonatal intensive care unit with jaundice, hypotonia and convulsive phenomena during hypoglycemia and fed via nasogastric tube. She was discharged after 36 days. Cerebral CT Scan and brain ultrasound at birth were normal. At the age of 9 months, she suffered from two episodes of pneumonia. Early motor milestones were severely delayed: she acquired the sitting position at the age of 3.5 years and crawled at the age of 6 years. Her language was limited to rare vocalizations and never developed further. At the age of 3 years, she experienced epileptic seizures characterized by impairment of consciousness and head hypotonia, initially treated with Phenobarbital and subsequently with Sodium Valproate plus Nitrazepam achieving a completely seizure-free period until the age of 16 years when seizures, hemiclonic during wakefulness (right side) and tonic during sleep, recurred. At the age of 16 years, her height was 150 cm (<3rd centile), weight 28 kg (<3rd centile) and head circumference 52 cm (3rd centile). At the same age, EEG showed epileptiform abnormalities, more frequently on fronto-temporal areas of the left hemisphere. A tonic seizure was recorded during polysomnography at 20 years of age. (Fig. 2a).

The subsequent increases in dosage of both Sodium Valproate *and* Clobazam led to the disappearance of the phenomena during wakefulness. Tonic seizures during sleep recurred with monthly frequency during her life. At the age of 26 years, EEG record showed irregular background activity characterized by abnormalities and spike-wave complexes, more evident during drowsiness and sleep (Fig. 2b). At the age of 17 years, she showed severe scoliosis followed by reduced thorax expansion and dysventilation with respiratory insufficiency. Her pubertal development at the age of 17 years was M2 P2; first menses begun at the age of 19 years but then she presented amenorrhea. During her entire life she suffered from episodes of gastro-esophageal reflux and dysphagia. After percutaneous endoscopic gastrostomy (PEG) placement at the age of 19 years, the frequency of respiratory infections (*ab ingestis* pneumonia) decreased and she gained weight (8 Kg). On ophthalmological examination, convergent strabismus was detected.

Facial dymorphisms consisted of dolichocephaly, high forehead, long face, hypertelorism, epicanthic fold, anterverted nares, micrognathia, high-arched palate, abnormal dentition (oversized and overlapping incisors) and gum hypertrophy, open-tended mouth and myopathic face (Fig. 1a–f). She was nonverbal and non-ambulatory. In particular she presented mild scoliosis, tetraparesis, pectus excavatum, microsplanchnia, reduced subcutaneous fat. Abdomen ultrasound scan, spinal X-ray, ECG, Auditory Brain Responses (ABR) and brain MRI were normal. The patient died of respiratory distress at 26 years.

**Fig. 3:**
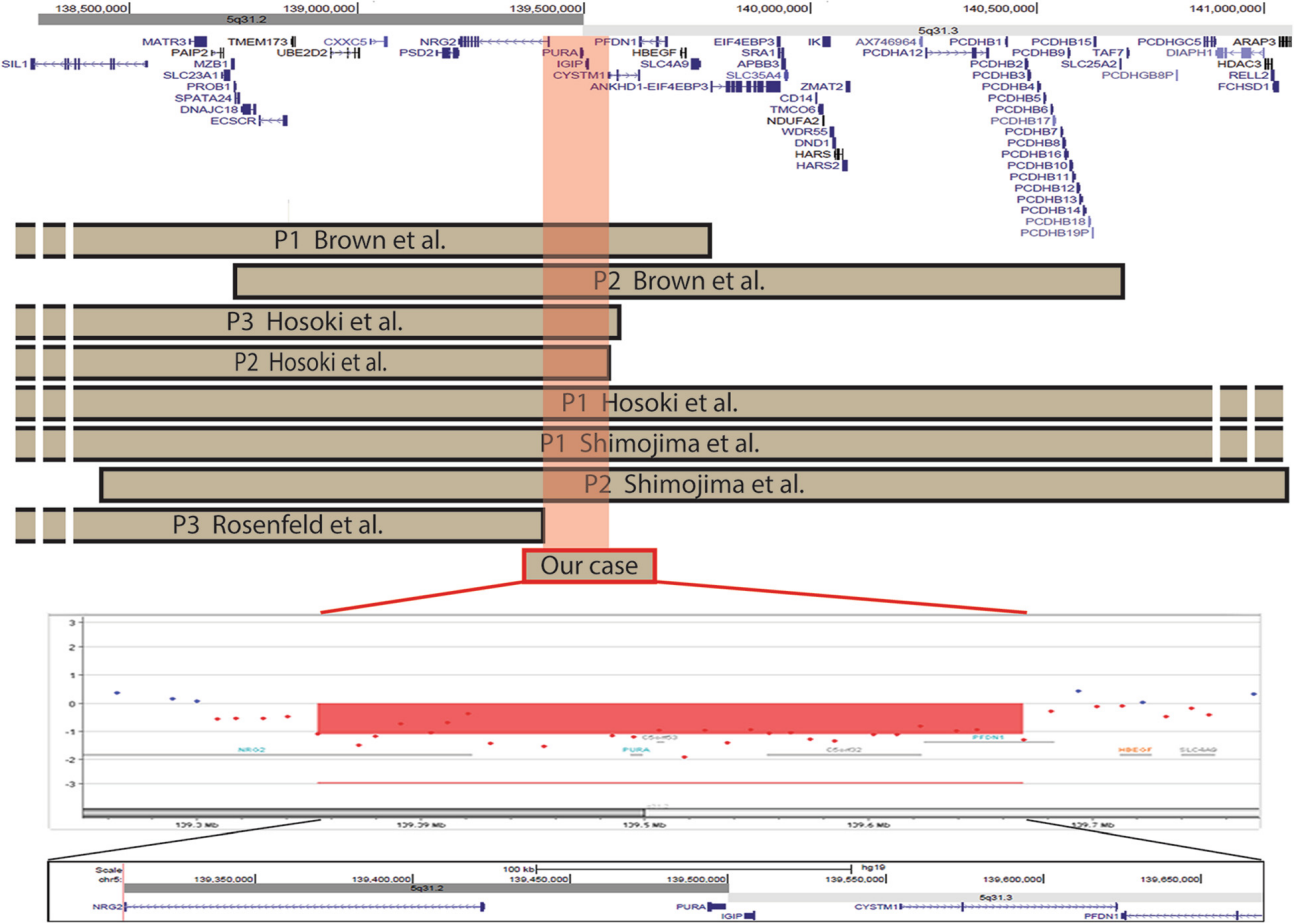
Schematic representation of our patient’s deletion compared with previously reported patients. *Top*. The screenshot spans 2.5 megabases of chromosome 5q13.2-q13.3. UCSC genes (GRCh37/hg19) are shown. *Middle*. Brown bars indicate *de novo* deletion of patients reported in the literature that have been characterised by molecular cytogenetics. Our case is represented by a red bar. The light red box indicates the common deleted region of ~101 Kb among patients sharing the 5q31.3 microdeletion syndrome phenotype. *Bottom*. Magnified view of breakpoint boundaries detected by array-CGH analysis using a 180 k Agilent kit. The deleted regions aligned with the UCSC map (hg19) are shaded in red

In the absence of any etiological diagnosis, array-CGH (Agilent Human Genome CGH Microarray Kit 180 k) analysis revealed a copy number loss of ~360 kb at 5q31.2q31.3 (Fig. 3). No additional rare copy number changes were detected in her genome. The deleted genomic region, according to the GRCh37/hg19, included *PURA* (located in chromosomal region 5q31.2), *IGIP* and *CYSTM1* (both located at 5q31.3), while the proximal and distal deletion breakpoint encompassed neuregulin 2 (*NRG2*, located at 5q31.2) and prefoldin subunit 1 (*PFDN1*, located at 5q31.3) genes, respectively (Fig. 3). Real-time quantitative PCR (qPCR) assays, performed on DNA from the patient and her parents, demonstrated that 5q31.2q31.3 deletion originated *de novo* (data not shown). The final interpretation of the rearrangement was arr[hg19] 5q31.2q31.3(139,299,777x2,139,308,862-139,669,265x1,139,681,675x2) dn.

## Discussion

To date at least seven affected children carrying a 5q31.2q31.3 deletion, varying in size from 2.6 to 5 Mb, have been reported [1–4]. The present patient, aged 26 years, is the oldest reported individual with the smallest 5q31.2q31.3 microdeletion of 360 kb (Fig. 3). The minimal critical region of overlap between all published cases [1–4], including the present one, is around ~101 kb and spans three genes: *PURA*, *IGIP*, *CYSTM1* [3] (Fig. 3). While the function of *IGIP* and *CYSTM1* is still unknown, *PURA* has been proposed earlier as a candidate gene [1], since it encodes Pur-α, a highly conserved multifunctional protein that has an important role in normal postnatal brain development in animal models [6, 7].

In addition, *de novo* heterozygous *PURA* mutations, including missense and truncating mutations, were recently identified in several subjects with the neurodevelopmental phenotype of 5q31.3 deletion syndrome [5, 8, 9]. Different mechanisms based on mutation type have been postulated: either a dominant negative effect or functional haploinsufficiency in truncating mutations [8], and a negative effect on protein function in missense mutations [5].

The clinical history of our patient indeed summarizes the mainly neurodevelopmental phenotype described in children with 5q31.3 microdeletion syndrome [1–4]. The perinatal period was characterized by neonatal hypotonia associated with severe feeding difficulties and respiratory distress. In our patient, feeding difficulties were severe and only percutaneous endoscopic gastrostomy (PEG) placement allowed her to gain weight and reduce the frequency of respiratory infections (*ab ingestis* pneumonia). Almost all reported affected children both with 5q31.2q31.3 deletions [1–4] and *PURA* mutations [5, 8] were non-ambulatory at the time of evaluation, as observed in our patient, who showed a more severe outcome consisting of tetraparesis at the age of 8 years. Respiratory distress, reported in several paediatric patients with 5q31.2q31.3 deletion [1–4] or *PURA* point mutation [5, 8], was complicated in our patient by the additional development of severe scoliosis and thoracic deformity since age of 15 years.

Recognizable craniofacial features similar to previously described cases, including dolichocephaly, long face, hypertelorism, micrognathia, high-arched palate, open-tended mouth, myopathic face were retained in our patient (Fig. 1a–f) highlighting that in 5q31.2q31.3 microdeletion patients facial appearance is an important diagnostic clue.

In contrast to previous reported patients, our case did not show any signs of hypomyelination or myelin maturation delay or other anomalies at brain MRI, while an abnormal EEG [1–5, 8] was documented from birth. Her EEG findings, initially characterised by global slow background activity, prompted early treatment with anti-epileptic medication that allowed to maintain our patient seizure-free during wakefulness, while tonic seizures during sleep remained at low frequency until her death. Interestingly, a recent study on model organism *Ceanorhabditis Elegans* has shown that homozygous *plp1* mutants were sterile with absence of oocytes, suggesting a requirement for *plp1* in germline differentiation [5]. It has been proposed that the human *PURA* ortholog might have an essential role in both somatic and germline tissues [5]. Since Human *PURA* is expressed both in brain and ovarian tissues [5], we hypothesize that *PURA* haploinsufficiency may be also associated with the delayed pubertal development and amenorrhea observed in our patient, suggesting that this clinical signs might be a part of the PURA-related disorder in female patients.

## Conclusion

The clinical and molecular findings reported here provide further evidence that patients with a the 5q31.3 microdeletion syndrome is a clinically discernible *PURA*-related disorder. Our patient exhibited a remarkable deterioration of clinical symptoms starting at the beginning of adolescence. While epileptic seizures were successfully treated during her life, feeding problems showed a poor outcome. Her respiratory problems increased and eventually became severe enough to cause her death. This report, the first long-term follow-up of a patient with 5q31.2q31.2 microdeletion involving *PURA*, will help to define the syndrome’s clinical evolution and complications, and suggests that a long term neurological and EEG follow-up, as well as feeding and respiratory care, will help to improve the quality of life of patients with this condition.

## Material and methods

### Array-CGH and quantitative (qPCR) analysis

DNA was prepared from peripheral blood using standard procedures and obtained after informed consent. Array-CGH analysis was performed using the Agilent array 180 K (Agilent Technologies) according to the manufacturer’s protocol. Data analysis was performed using Agilent Cytogenomics Ed 2.5.8.1. Oligo positions are referred to the UCSC Genome Browser (Feb 2009 assembly, hg19).

Real-time quantitative PCR (qPCR) assays was performed on DNA from the patient and her parents using SYBR Green and analysed on an ABI PRISM 7900HT sequence detection system (Applied Biosystems, Foster City, CA). Probe locations were: RT1, chr5:139419393–139419453 (hg19) and RT2, chr5:139619433–139619490 (hg19).

## Consent

Written informed consent was obtained from patient’s parents for the publication of this report and any accompanying images.
